# Editorial Notes, News and Miscellany

**Published:** 1913-04

**Authors:** 


### Editorial Notes, News and Miscellany.

THE great alienist and author, Dr. Allen McLane Hamilton, is dead.

THE JOURNAL is unavoidably late this month by reason of press of Legislature work at the printing office.

THE IRONY OF FATE.—"Some ha' meat and canna eat, and ithers canna get it." J. P. Morgan died of starvation.

WANTED: A man to undertake the sale of patent medicines. The advertiser guarantees that it will be profitable to the undertaker.—*Ex.*

DR. A. H. EVANS, of Eagle Pass, has been appointed collector of customs at that place. Dr. Evans was a long time quarantine officer there and is well known to, and popular with, the physicians of Texas. Congrats.—D.

AFTER THE OCTOPUS.—Elsewhere is mentioned a suit against the A. M. A. for $500,000, filed by a New York medical college, with Charles J. O'Connor pushing it. Now a doctor, John A. Hawkins, of Pittsburg, Pa., files suit for libel in publishing that he had gone blind and could no longer practice medicine. Truly, the way of the transgressor is hard.

THE LEGISLATURE authorized by enactment the confinement of habitual drunkards in the insane asylum for treatment. They are to be "tried" by a jury and committed as are lunatics under our absurd law. They also abolished the gallows and substituted electrocution for State killings in future. They also made "pistol toting" a felony. Later: Vetoed.

HEDIOSIT ($C_7H_{12}O_7$).—A pleasant tasting nutrient and sugar substitute for diabetes is being presented to physicians by the Farbwerke-Hoechst Company, 34 Beach street, New York, in the form of Hediosit, a chemical compound which is meeting with much favor in Europe. It is easily oxidized by the worst diabetic cases and is utilized as food. In addition, it possesses the unique property of considerably reducing the excretion by diabetic patients of the sugar derived from other sources. There is no increase in glycosuria, even in the worst cases, and ordinarily it is greatly reduced. Hediosit is an important adjunct to the dietetic treatment of diabetes and offers great relief to the sufferer.

---

Facts About Phylacogens.—Practitioners who have a fondness for figures, and who want definite, first-hand knowledge of what the Phylacogens are accomplishing in the way of actual clinical results are urged to turn to the display announcement in the current issue of this journal bearing the signature of Parke, Davis & Co. Here, under the title of "The Value of the Phylacogens," one finds the results in 4148 cases of infectious diseases that have been treated with Phylacogens. One also reads in detail what is credited to each individual Phylacogen. For instance, you may be interested in rheumatic affections. You see at a glance that a certain number of cases have been treated and reported; the same glance tells you how many of them were treated successfully. This is equally true of pneumonia cases, erysipelas cases, gonorrheal cases, mixed-infection cases. Figures are apt to be tiresome. These figures are not so; they tell what every practitioner of medicine wants to know or should know. We commend the announcement to our readers.

---

DR. FRIEDMAN AND THE NEW YORK DOCTORS.—If the accounts given in the newspapers of the treatment accorded Dr. Friedman by the hospital doctors and health officers in New York are true, it is most reprehensible and almost unbelievable. An opportunity to demonstrate his alleged cure before the authorities is demanded by every consideration of humanity, professional courtesy and fair play. And that any red tape, or technicality, or feeling of envy, or jealousy, should stand in the way is inconsistent with the American boast of a square deal. Particularly contemptible was the refusal to admit to the Bellevue Hospital for Dr. Friedman's treatment a consumptive physician from another State, who, according to news dispatches, walked miles through snow to reach a train to take him to New York, because he was not a citizen of New York, and the pretext that Dr. Friedman did not have a license to practice is puerile in the extreme and worthy only of contempt.

The Texas Legislature passed a resolution inviting Dr. Friedman to come to Texas to treat consumptives, and his reply was that he would do so if the Federal authorities would let him. We will treat him right.

NEAR LEGISLATION.—The Legislature adjourned April 1. Not a bill passed that was of interest to the medical profession, unless I except that which dissolved the Tuberculosis Commission and created a board of trustees to manage the Carlsbad sanitarium for indigent consumptives. Yes, I should state that a bill to prevent the pollution of streams—a matter of the very first importance—was passed, but it exempted all packing houses. Everybody knows that a big packing house could not pollute a river if it tried, being disinfected by all means known to science, deodorized by camphor and perfumed with attar of roses. They can do no harm. The bill for a lunacy commission got lost in the shuffle; that creating an institution for the feeble-minded passed and Colquitt vetoed it; the marriage bill never saw daylight; and, as stated, the vasectomy bill was killed. That means more poorhouses, more jails, more reformatories, more asylums and a heavier burden for the taxpayers. The optometry bill was killed. The bill to prevent peddling patent medicines and the bill for a board of examiners to license masseurs also turned up their toes and went to sleep. Here is the list of immortals who voted to perpetuate the multiplication of the defective classes and build palaces for the care of their progeny: Senators Morrow, Kauffman, Nugent, Wiley, Brelsford, Westbrook, Conner, Cowell, Hudspeth, McNealus, Murray, Terrell, Townsend, Watson.

Absent: Bailey, Gibson, Greer.

Paired: Darwin, "yes"; McGregor, "no."

